# Infection profile of patients undergoing autologous bone marrow transplantation in a Brazilian institution

**DOI:** 10.1590/S1516-31802012000100003

**Published:** 2012-02-13

**Authors:** Kelli Borges Santos, Abrahão Elias Hallack, Girlene Alves Silva, Angelo Atalla, Marcus Matta Abreu, Luiz Cláudio Ribeiro

**Affiliations:** I MSc. Assistant Professor, School of Nursing, Universidade Federal de Juiz de Fora (UFJF), Juiz de Fora, Minas Gerais, Brazil.; II PhD. Adjunct Professor, Department of Clinical Medicine, Universidade Federal de Juiz de Fora (UFJF), Juiz de Fora, Minas Gerais, Brazil.; III PhD. Adjunct Professor, School of Nursing, Universidade Federal de Juiz de Fora (UFJF), Juiz de Fora, Minas Gerais, Brazil.; IV MSc. Adjunct Professor, Department of Clinical Medicine, Universidade Federal de Juiz de Fora (UFJF), Juiz de Fora, Minas Gerais, Brazil.; V MD. Specialist in Thoracic Surgery and Professor of Surgery, Faculdade de Ciências Médicas de Juiz de Fora (FCMS/JF), Juiz de Fora, Minas Gerais, Brazil.; VI PhD. Demographer and Associate Professor, Department of Statistics, Universidade Federal de Juiz de Fora (UFJF), Juiz de Fora, Minas Gerais, Brazil.

**Keywords:** Hematopoietic stem cell transplantation, Transplantation, autologous, Infection, Risk factors, Infection control, Transplante de células-tronco hematopoéticas, Transplante autólogo, Infecção, Fatores de risco, Controle de infecções

## Abstract

**CONTEXT AND OBJECTIVE:**

Hematopoietic stem cell transplantation (HSCT) has been widely used for treating oncological and hematological diseases. Although HSCT has helped to improve patient survival, the risk of developing infection during hospitalization is an important cause of morbidity and mortality. This study aimed to analyze the infection profile during hospitalization and the associated risk factors among patients undergoing autologous HSCT at the University Hospital, Universidade Federal de Juiz de Fora.

**DESIGN AND SETTING:**

This was a cross-sectional study on patients undergoing autologous HSCT at a public university hospital.

**METHODS:**

Patients with febrile neutropenia between 2004 and 2009 were retrospectively evaluated re garding their infection profile and associated risk factors.

**RESULTS:**

Infection occurred in 57.2% of 112 patients with febrile neutropenia. The main source of infec tion was the central venous catheter (25.9%). Infection was chiefly due to Gram-positive bacteria, although Gram-negative-related infections were more severe and caused a higher death rate. Sex, age, skin color, nutritional status and underlying disease were not associated with the development of infection. Patients with severe mucositis (Grades III and IV) had a higher infection rate (P < 0.001). Patients who developed pulmonary complications during hospitalization had higher infection rates (P = 0.002). Infection was the main cause of death (57.1%) in the study sample.

**CONCLUSION:**

Strategies aimed at reducing infection-related mortality rates among patients undergoing autologous HSCT are necessary.

## RESUMO

**CONTEXTO E OBJETIVO:**

O transplante de células-tronco hematopoiéticas (TCTH) vem sendo ampla mente utilizado no tratamento das doenças onco-hematológicas. Embora o TCTH tenha colaborado para a melhora na sobrevida dos pacientes, o risco de desenvolver infecção no período de internação é uma importante causa de morbi-mortalidade. O presente estudo teve como objetivo analisar o perfil das in fecções no período de internação e os fatores de risco associados entre os pacientes submetidos ao TCTH autólogo, no Hospital Universitário da Universidade Federal de Juiz de Fora.

**TIPO DE ESTUDO E LOCAL:**

Trata-se de um estudo transversal sobre pacientes submetidos a transplante autólogo, em um hospital público universitário.

**MÉTODOS:**

Foram analisados retrospectivamente os pacientes que apresentaram neutropenia febril no período de 2004 a 2009, com relação ao perfil infeccioso e os fatores de risco associados.

**RESULTADOS:**

A infecção foi determinada em 57,2% dos 112 pacientes com neutropenia febril. A prin cipal fonte de infecção foi o cateter venoso central (25,9%). A infecção ocorreu principalmente devido a bactérias Gram-positivas, apesar de as infecções causadas por bactérias Gram-negativas terem sido mais graves e causado maior taxa de morte. Sexo, idade, cor da pele, estado nutricional e doença de base não estiveram associados com o desenvolvimento da infecção. Pacientes com mucosite grave (graus III e IV) apresentaram maior taxa de infecção (P < 0.001). Os pacientes que desenvolveram complicações pulmo nares durante a internação apresentaram maiores taxas de infecção (P = 0,002). A infecção foi a principal causa do óbito (57,1%) na amostra estudada.

**CONCLUSÃO:**

São necessárias estratégias voltadas para a redução da taxa de mortalidade relacionada com infecção entre pacientes submetidos ao TCTH autólogo.

## INTRODUCTION

Hematopoietic stem cell transplantation (HSCT) has been widely used for treating oncological and hematological diseases. Although its use has increased patient survival, the risk of infec tion is an important cause of morbidity and mortality among those undergoing this therapeutic approach.^1^

In spite of lower infection rates with autologous bone marrow transplantation (BMT), in comparison with allogeneic BMT, infec tion is the second most frequent cause of death among patients under going autologous BMT, and is second only to disease relapse.^2^^,^^3^

Patient who have undergone autologous BMT are at higher risk of infection during the neutropenia period, and for up to 30 days after bone marrow grafting. The neutropenia period and breach of the mucocutaneous barrier are the main risks for devel opment of infections.^4^^,^^5^

Hospital-acquired infection rates among HSCT patients depend on disease severity, drugs used and invasive procedures performed.^6^ Furthermore, the more severe the neutropenia is, the more frequent and aggressive the infections are.^4^

## OBJECTIVE

Taking into account the susceptibility of autologous BMT patients to infection during the neutropenia period, we assessed the infectious events occurring during hospitalization and their associated risk factors.

## METHODS

One hundred and fifteen patients underwent autologous BMT at the University Hospital, Universidade Federal de Juiz de Fora (UFJF), between 2004 and 2009. Of these, 112 patients developed febrile neutropenia, and were analyzed. This study was approved by the research ethics committee of the University Hospital, UFJF.

All patients received antiviral prophylaxis consisting of acy clovir 240 mg/m2/day, divided into four doses, until they received the graft, and also received granulocyte colony-stimulating factor (G-CSF), 5 mg/kg/day, in accordance with our local protocol.

Three blood samples were obtained for blood cultures: one from each line of the central venous catheter and one from peripheral blood. Empirical treatment with cefepime (4 g/day, divided into two doses) was instituted for every neutropenic patient with fever.

Febrile neutropenia was considered to be present when ever a patient with a neutrophil count below 500/mm3, or below 1,000/mm3 but predicted to fall to below 500/mm3,7 developed a single axillary temperature reading ≥ 38 °C, or two readings ≥ 37.5 °C within a 12-hour period. Cultures of samples obtained from different sites were performed whenever a specific infec tious site was suspected.

After three days of treatment, the patients were reassessed and, if the fever persisted, a new blood culture was performed. The empirical therapeutic regimen was adjusted in accordance with the recommendations of the Centers for Disease Control and Prevention (CDC).^8^

When blood cultures and cultures from the catheter tip yielded the same organism, with a higher number of colony-forming units (CFU) in the latter, and when the catheter tip culture grew more than 15 CFU, the infection was considered to be catheter-related.

For univariate analysis, the chi-square or Fisher exact test was used to determine the association between infection and risk factors (sex, age, underlying disease, duration of neutropenia, drugs used in the conditioning regimen, presence of mucositis, pulmonary compli cation, diarrhea and sinusoidal obstructive syndrome). Logistic regres sion was used for multivariate analyses, with the inclusion of variables that presented P values < 0.05 on univariate analysis. The Statistical Package for the Social Sciences (SPSS) 13.0 software was used for cal culations, and P values < 0.05 were considered statistically significant.

## RESULTS

Out of the 115 patients who underwent autologous BMT, 112 developed febrile neutropenia (97.39%). The median age of this latter group was 43 years (range: 8-69 years). Table 1 shows the characteristics of the study population. 

The neutropenia period ranged from four to 25 days (median: nine days). Multiple myeloma patients remained neutropenic for seven days, on average, while those with lymphoma remained neutropenic for 12 days, on average (P < 0.001). Although the difference between the groups was significant, the duration of neutropenia was not associated with occurrences of infection (P = 0.323). There was no impact on the number of infused cells on occurrences of infection (P = 0.129).

Six out of 60 patients with positive blood culture had more than one microorganism. Table 2 shows the microorganisms yielded in the first and second blood cultures. One hundred and four different microorganisms were isolated, and the most fre quent ones were: coagulase-negative Staphylococcus (24.3%), Staphylococcus aureus (13%), Klebsiella pneumoniae (12.1%), Escherichia coli (9.7%), Pseudomonas aeruginosa (4.3%) and Enterobacter cloacae (4.3%). There were also isolates of fungi (Candida parapilosis, 0.9%; Candida albicans, 5.2%; and Fusarium sp., 0.9%) and intestinal parasites (Cryptosporidium spp., 1.7%; Isospora sp, 0.9%; and Strongyloides stercoralis, 2.6%).

Eighty-five instances of infection were identified in 63 patients (57.2% of the study population), of whom 22 (34.9%) had a sec ond source of infection. Table 3 shows the sources of infections identified in the study population.

The catheter was the main infection source identified in the study population, and accounted for the infections of 29 patients (25.9% of the study population). Catheter-related infec tion was caused by Gram-positive bacteria in 48.3% of the cases (P < 0.001).

An analysis was made between different risk factors and occurrences of infection. Two cases were taken to be missing data, since it was not possible to determine the occurrence of infection. Table 4 shows the results from this analysis.

Among the 63 patients for whom some kind of microorgan ism was identified as the cause of their infection, eight (12.6%) had fungal infection (Candida albicans), 30 (47.6%) had Gram positive bacteria and 25 (39.6%) had Gram-negative bacteria. Viral infection due to cytomegalovirus was identified by means of the polymerase chain reaction (PCR) in only one patient.

**Table 1.** Characteristics of the study population (n = 112)

Characteristics	Frequency (n)	Percentage (%)
Sex		
Male	64	57.1
Female	48	42.9
Associated comorbidity		
Present	37	33.0
Absent	75	67.0
Underlying disease		
Multiple myeloma	57	50.9
Hodgkin’s lymphoma	32	28.6
Non-Hodgkin’s lymphoma	18	16.1
Neuroblastoma	02	1.8
Acute myeloid leukemia	03	2.7
Conditioning		
Cyclophosphamide, carmustine and etoposide	48	42.9
Melphalan	57	50.9
Carmustine, etoposide, cytarabine and melphalan	5	4.5
Others	2	1.8
Total	112	100

**Table 2.** Frequency of microorganisms isolated from blood cultures

Microorganism	Number of cases in first blood culture	%	Number of cases in second blood culture	%	Total number of cases	% total
Gram-positive bacteria						
*Coagulase-negative Staphylococcus*	23	20.0	3	2.6	23	20.0
*Staphylococcus aureus*	9	7.8	5	4.3	11	9.5
*Streptococcus alpha haemolyticus*	1	0.9	-	-	1	0.9
Total	32	27.8	8	6.9	34	29.5
Gram-negative bacteria						
*Enterobacteriaceae family*						
*Klebsiella pneumoniae*	13	11.3	-	-	13	11.3
*Escherichia coli*	5	4.3	1	0.9	6	5.2
*Enterobacter cloacae*	5	4.3	-	-	5	4.3
*Serratia marcescens*	1	0.9	-	-	1	0.9
*Non-fermenting*						
*Acinetobacter baumanii*	2	1.7	-	-	2	1.7
*Pseudomonas aeruginosa*	3	2.6	-	-	3	2.6
*Non-identified non-fermenting gram-negative bacteria*	-	-	1	0.9	1	0.9
Total	29	25.2	2	1.7	31	26.9
Fungi						
*Candida parapilosis*	1	0.9	1	0.9	1	0.9
Total	1	0.9	1	0.9	1	0.9
Total (all microorganisms)	62	53.9	11	10.4	66	57.3

**Table 3.** Sources of infection

Characteristics	Frequency (n)	Percentage (%)
Catheter-related infection	29	34.1
Oral candidiasis	24	28.2
Pneumonia	10	11.7
Bacteremia	05	5.8
Urinary infection	05	5.8
Skin infection	04	4.7
Intestinal infection	04	4.7
Systemic fungal infection	02	2.3
Vaginal candidiasis	02	2.3
Total	85	100

**Table 4.** Factors associated with infection during hospitalization^*^

Features analyzed	Total	Presence of infection	%	P
Conditioning				
Cyclophosphamide, carmustine and etoposide	46	25	54.3	0.600
Melphalan	56	32	57.1	
Carmustine, etoposide, cytarabine and melphalan	6	5	83.3	
Others	2	1	50.0	
Underlying disease				
Lymphomas	48	27	56.3	0.575
Myeloma	57	32	56.1	
Others	5	4	80.0	
Associated comorbidity				
Present	37	26	70.3	0.050
Absent	73	37	50.7	
Mucositis				
Present	59	39	66.1	0.044
Absent	51	24	47.1	
Degree of mucositis				
I	24	11	45.8	0.020
II	19	14	73.7	
III	14	12	85.7	
IV	2	2	100.0	
Diarrhea				
Present	72	39	54.2	0.365
Absent	38	24	63.2	
Sinusoidal obstruction syndrome				
Present	6	4	66.7	0.632
Absent	104	59	56.7	
Pulmonary complications				
Present	15	14	93.3	0.002
Absent	95	49	50.0	
Sex				
Female	47	30	63.8	0.230
Male	63	33	52.4	

*Missing: two patients without data on infection.

Oral candidiasis was associated with higher grades (III and IV) of mucositis (P < 0.001). Two patients developed fungemia: one due to *Candida parapilosis* and one due to *Fusarium* spp. Fungi (*Cryptosporidium* spp. and *Isospora* spp.) were isolated from the stools of two patients.

*Mycobacterium tuberculosis* grew in a culture from secretions obtained from a cutaneous abscess. Out of the 104 microorganisms isolated, nine (8.6%) were cefepime-resistant. Although three (21.4%) of the *Staphylococcus aureus* isolates were methicillin-resistant (MRSA), they were uniformly sensitive to vancomycin. Blood cultures from six patients presented oxacillin-resistant coagulase-negative *Staphylococcus*.

Among the eight patients who died of infection, three (37.5%) presented Gram-negative bacteria in their cultures, one had *Fusarium spp.* and four had no growth in the cultures performed.

### Infection-associated factors

Mucositis (P = 0.04) and pulmonary complications (P = 0.002) during hospitalization were the only variables with a statistically significant impact on the occurrence of infection, on univariate analysis. Patients with higher grades (III and IV) of mucositis developed infections more frequently (P = 0.02). 

Patients with associated comorbidities, such as diabetes mellitus and systemic arterial hypertension, were more likely to develop infection, although this was not statistically significant (P = 0.05).

Both variables that showed statistical significance in relation to infection on univariate analysis (mucositis and pulmonary complications) were included in the logistic regression models. The results from this model are shown in Table 5. Only pulmonary complications remained an independent risk on multivariate analysis (P = 0.015), with an odds ratio of 13. Although the confidence interval of the odds ratio for the variable of pulmonary complications was large, this was due to the low frequency of events. Nonetheless, 14 out of the 15 individuals who had pulmonary complications developed infections, which shows that there was a strong association between these variables. Even though the P value related to mucositis was slightly above 5%, stratified analysis showed that among individuals who did not have pulmonary complications, the infection rate was significantly higher among patients who developed mucositis (P = 0.032).

**Table 5.** Multivariate analysis with risk factors for infection

Variables	P-value	Odds ratio	95% confidence interval	
			Lower	Upper
Mucositis	0.054	2.21	0.98	4.94
Pulmonary complications	0.015	13.23	1.65	105.98

*Adjusted for mucositis and pulmonary complications.

## DISCUSSION

In this study, 57.2% of the population had an infection identified during hospitalization. According to published data from a single Brazilian center, the infection rate among a pediatric population undergoing allogeneic and autologous BMT was 58.5%.^9^ In a multicenter study undertaken in southern and southeastern Brazil, the infection rate was 55%.^2^ Although these values are similar to ours, the comparison is hampered because we studied patients undergoing autologous BMT alone.

Studies in other countries on patients undergoing autologous BMT have indicated infection rates ranging from 27.6% to 48.2%,^10^^,^^11^ i.e. lower than the rate that we found. Although not statistically significant, there was a difference in the occurrence of infection in relation to the first years covered by this study. This might be accounted for by the little experience of managing autologous BMT patients that existed in those early days, which may have led to infection rates that were higher than those in the literature. 

In disagreement with the literature,^5^^,^^12^^,^^13^ age was not associated with the risk of infection in our study. This finding was possibly due to the small number of children in our sample. 

In agreement with the findings of Poutsiaka et al.,^14^ the length of the neutropenia period was not significantly associated with higher rates of infectious complications in our study. The same was observed in the study by Ninin et al.,^15^ in which patients undergoing autologous and allogeneic BMT had similar infection rates.

Although not statistically significant, multiple myeloma patients had the lowest infection rates, a finding that is similar to the data reported by Meyer et al.^16^ According to Gil et al.,^10^ acute myeloid leukemia patients undergoing autologous BMT have the highest risk of infection, since they remain neutropenic for longer periods. In our study, there was no association between the conditioning regimen and infection. 

The most common infection source was the catheter (34.1%). Our data were very close to those of Nucci and Maiolino,^2^ who reported a catheter-related infection rate of 38% in their patients. The associated factors may include: handling frequency, insertion site, contamination during insertion, skin colonization around the insertion site, contamination of the connection device between the infusion system and vascular access, contamination of the infusion fluid, contamination of solutions used to ensure catheter permeability and presence of distant infectious sites with hematogenic spread of infection.^4^^,^^8^

We also made this observation, such that patients with mucositis of grades III and IV had higher infection rates. 

Infection-related pulmonary complications have been considered to be important determiners of morbidity and mortality. According to Meyer et al.,^16^ pneumonia was the second most frequent cause of infection in these patients, occurring in 7.8% of the cases. According to Dettenkofer et al.,^6^ pneumonia is the most frequent infection in patients undergoing autologous BMT. 

Three patients had oxacillin-resistant *Staphylococcus aureus*. This microorganism increases the infection-related mortality rate of grafted patients, and has been isolated with higher frequency, in different places, over the past few years.^17^ No vancomycin-resistant microorganism was isolated in our study.

In accordance with the Brazilian and foreign literature, Gram-positive bacteria were the most frequent isolates in our study. Nonetheless, a significant increase in the number of infections due to Gram-negative bacteria has been reported over the past few years.^1^^,^^7^^,^^12^^,^^18^^,^^19^^,^^20^^,^^21^ This may be accounted for by greater use of prophylactic antimicrobials, chiefly quinolones.^22^

According to Laws et al.,^19^ Gram-negative bacterial infections are more severe and cause higher death rates, compared with Gram-positive bacterial infections. This may be explained by their greater virulence and resistance, and the latter is also due to their double cell membrane, which blocks the entry of some drugs.^23^

The most frequent isolate in our study was coagulase-negative *Staphylococcus*, in agreement with findings from different BMT centers.^6^^,^^15^^,^^16^^,^^19^^,^^24^^,^^25^ Until recently, these bacteria were considered contaminants, devoid of serious clinical importance.^26^ However, over the past few years, they have been acknowledged as important infectious agents. Catheters are the main means of entry for these microorganisms.^26^

Fungi were the second most frequently identified microorganisms in the infections we found. The main isolate was *Candida albicans,* and its presence was associated with mucositis. In autologous BMT, the likelihood of systemic candidiasis is very low, and occurs chiefly after the neutropenia period has resolved, or in the absence of mucositis.^27^

Similarly to findings from other centers,^2^^,^^8^^,^^11^^,^^14^infection was the main cause of death in our patients during hospitalization. It seemed to contribute towards longer hospital stay, although this association did not present statistical significance (P = 0.081).

## CONCLUSIONS

The infection rate found among patients undergoing BMT was high (57.2%), and the main source was the catheter (34.1%). We believe that continuing training programs and basic preventive measures, targeted to reduce infectious complications during the hospitalization of patients undergoing BMT, are paramount. Further studies are necessary before more effective measures to protect this infection-prone population can be implemented.
